# Supplementary figures and images for: Selective transport of fluorescent proteins into the phage nucleus
Source: PLoS One. 2021 Jun 10;16(6):e0251429. doi: 10.1371/journal.pone.0251429 (PMC8191949; doi:10.1371/journal.pone.0251429)

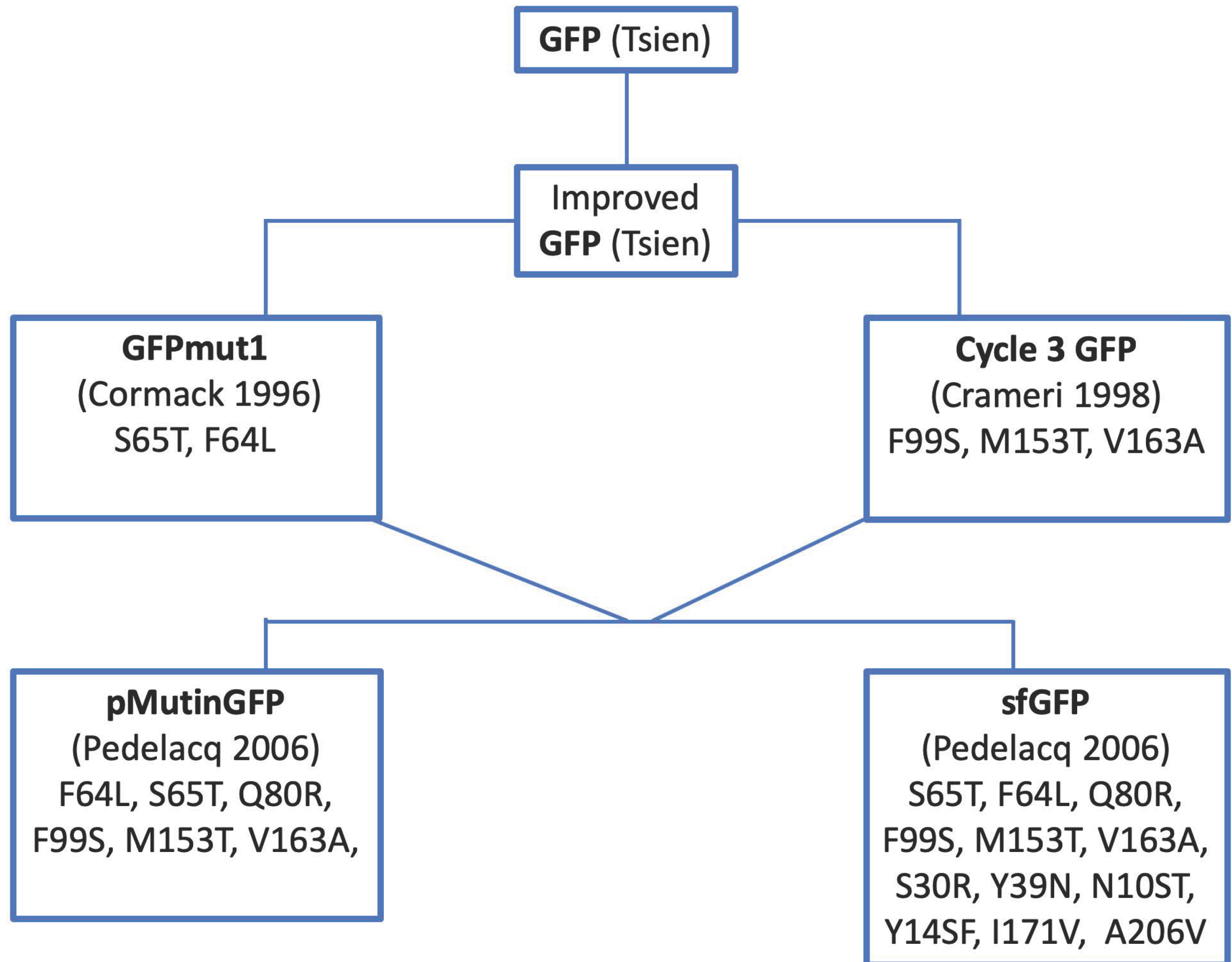

Supplement: S1 Fig — (PDF) [file pone.0251429.s001.pdf]

Figure S2

C

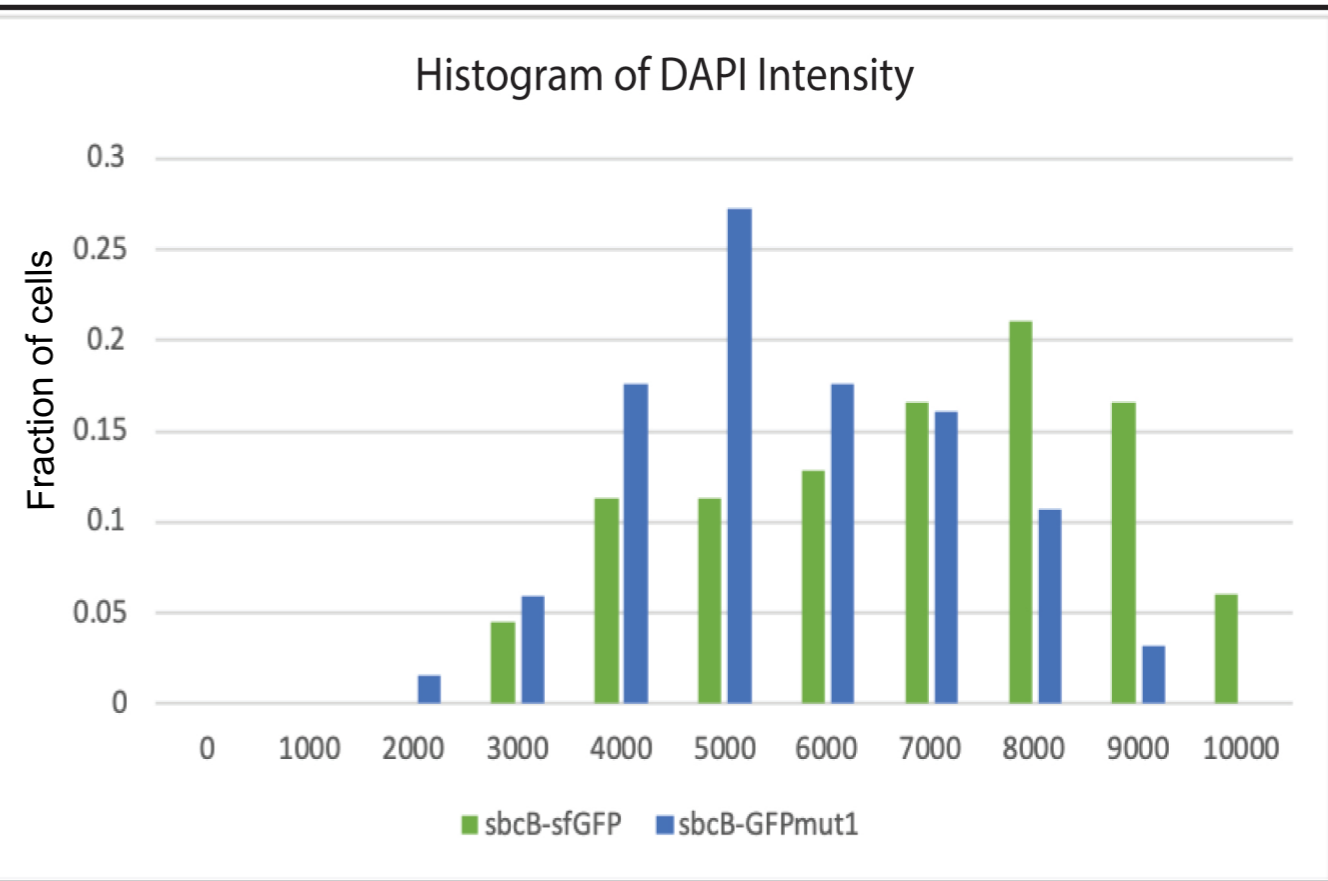

B

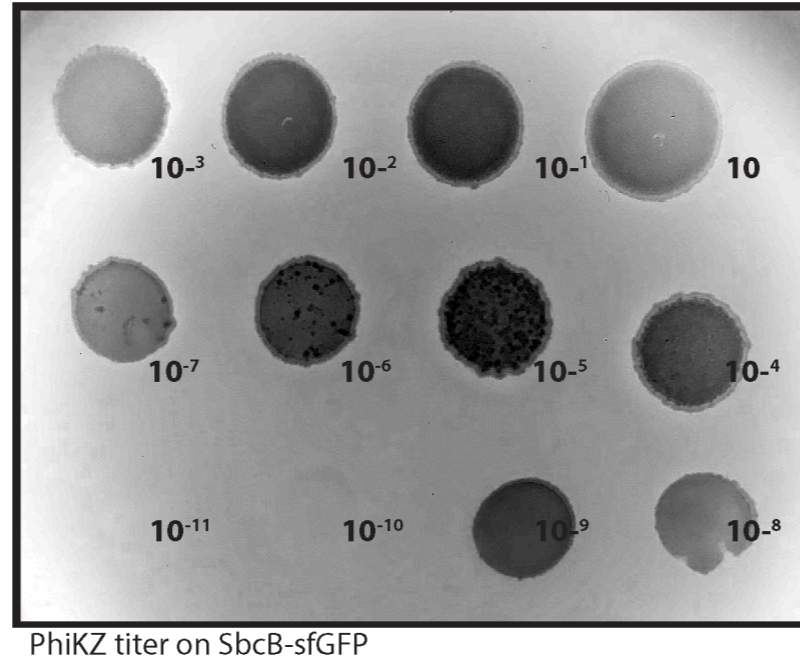

A

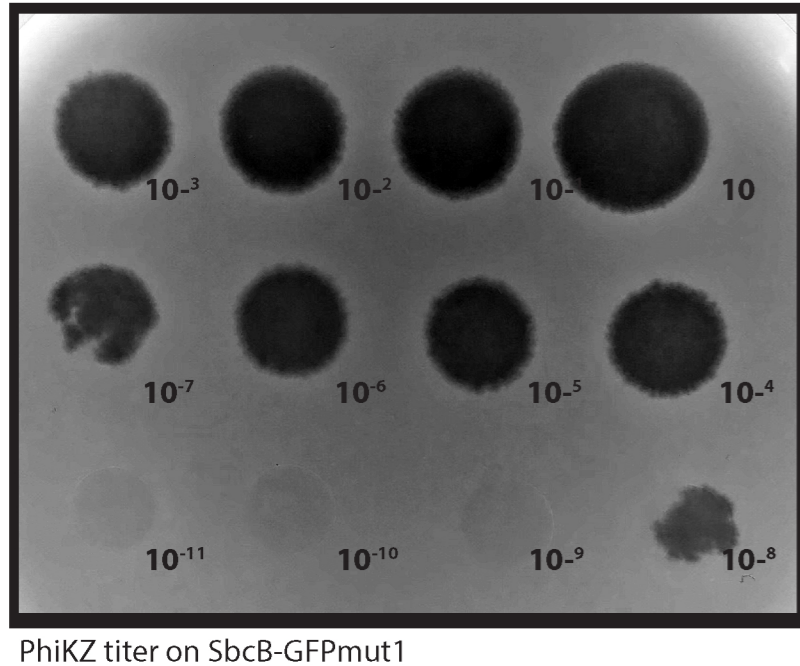

Supplement: S2 Fig — A. ΦKZ phage titer on a lawn of Pseudomonas aeruginosa expressing sbcB-GFPmut1. Titer, calculated at 2 x 10^11^ pfu/mL is reduced approximately 10-fold compared to (B). B. ΦKZ phage titer on a lawn of Pseudomonas aeruginosa expressing sbcB-sfGFP. Titer is calculated as approximately 2 x 10^12^ pfu/mL. C. A histogram of DAPI (DNA stain) intensity indicates that cells expressing sbcB-mut1 (blue columns, n = 187) have lower intensity, compared to cells expressing sbcB-sfGFP (n = 133). This suggests that DNA concentration is reduced by the presence of the host nuclease inside the phage nucleus. (PDF) [file pone.0251429.s002.pdf]
