# Supplementary material for: Selective transport of fluorescent proteins into the phage nucleus
Source: PLoS One. 2021 Jun 10;16(6):e0251429. doi: 10.1371/journal.pone.0251429 (PMC8191949; doi:10.1371/journal.pone.0251429)
Supplement: S1 Table — (DOCX) [file pone.0251429.s003.docx]

**S1 Table**

| **strain name** | **organism** | **plasmid** | **description** |
| --- | --- | --- | --- |
| K2733^1^ | *Pseudomonas aeruginosa* | none | PAO1 ΔMexAB–OprMΔMexCD–OprJΔMexEF–OprNΔMexXY |
| KN156 | *Pseudomonas chlororaphis* | pKN055 | mCherry from pRSET-mCherry |
| KN150 | *Pseudomonas chlororaphis* | pKN053 | GFPmut1 from pSG1729 |
| KN202 | *Pseudomonas chlororaphis* | pKN69 | sfGFP |
| KN210 | *Pseudomonas chlororaphis* | pKN70 | pMutin GFP+ |
| KN213 | *Pseudomonas chlororaphis* | pKN71 | CFP |
| KN336 | *Pseudomonas aeruginosa (PAO1)* | pKN112 | C-terminal sfGFP and cas3 from PA14 genome |
| KN339 | *Pseudomonas aeruginosa (PAO1)* | pKN113 | C-terminal GFPmut1 and cas3 from PA14 genome |
| KN517 | K2733 | pKN168 | C-terminal sfGFP and cas13 with crRNA to gp146 |
| KN519 | K2733 | pKN169 | C-terminal GFPmut1 and cas13 with crRNA to gp146 |
| KN546 | K2733 | pKN174 | C-terminal GFPmut1 and cas9 |
| KN548 | K2733 | pKN175 | C-terminal sfGFP and cas9 |
| KN573 | K2733 | pKN61 | N-terminal mCherry from pRSET fused to C-terminal GFPmut1 |
| KN574 | K2733 | pKN118 | N-terminal GFPmut1 -- C-terminal mCherry from pRSET |
| KN473 | K2733 | pKN152 | GFPmut1 F99S |
| KN474 | K2733 | pKN153 | GFPmut1 M153T |
| KN475 | K2733 | pKN154 | GFPmut1 V163A |
| KN524 | K2733 | pKN057 | C-terminal GFPmut1 and 50sL28 (rpmB) from PA01 genome |
| KN525 | K2733 | pKN70 | pMutin GFP |
| KN526 | K2733 | pKN151 | WTGFP |
| KN527 | K2733 | pKN71 | CFP |
| KN528 | K2733 | pKN055 | mCherry from pRSET-mCherry |
| KN529 | K2733 | pKN69 | sfGFP |
| KN569 | K2733 | pKN62 | C-terminal mCherry from pRSET and 50S ribosomal protein L28 from PA01 genome |
| KN513 | K2733 | pKN75 | C-terminal sfGFP and gp146 from PhiKZ genome |
| MAC140 | K2733 | PMAC11 | SbcB–sfGFP |
| MAC150 | K2733 | PMAC12 | sbcB-GFPm1 |
| VC_606 | K2733 | pVC119 | mChPA3PhuZ-B1/1 |

^1^Choudhury, D., Paul, D., Ghosh, A.S., Talukdar, A.D., Choudhury, M.D., Maurya, A.P., Dhar, D., Chakravarty, A., Bhattacharjee, A. Effect of single dose carbapenem exposure on transcriptional expression of blaNDM-1 and mexA in Pseudomonas aeruginosa*. Journal of Global Antimicrobial Resistance* 7, 72–77 (2016).
